# Regulatory, scientific, and ethical issues arising from institutional activity in one of the 90 Italian Research Ethics Committees

**DOI:** 10.1186/s12910-021-00605-7

**Published:** 2021-04-07

**Authors:** G. Benfatto, Regulatory Group, Ethics Committee Catania 1 (Group), F. Drago

**Affiliations:** 1 Clinical Pharmacology and Pharmacovigilance Unit, Regional Pharmacovigilance Center of Catania, G Rodolico-San Marco University Hospital, Catania, Italy; 2 Ethics Committee, Catania 1, G Rodolico-San Marco University Hospital, Via Santa Sofia 78, 95125 Catania, Italy

**Keywords:** Clinical trials, Placebo, Minors/Parental consent, Research Ethics Committees/consultation

## Abstract

**Background:**

This paper highlights the issues that one of the 90 Italian Research Ethics Committees (RECs) might encounter during the approval phase of a clinical trial to identify corrective and preventive actions for promoting a more efficient review process and ensuring review quality. Publications on the subject from Italy and the rest of Europe are limited; encouraging constructive debate can improve RECs’ service to the subject of the clinical trial.

**Methods:**

We retrospectively reviewed a cohort of 822 clinical trial protocols, initially reviewed by REC, from June 2014 to December 2018. Data collected for each protocol were type of trial, sample size, use of placebo, number and kind of revisions requested by the REC before approval, and time taken for approval. Data for each protocol were collected by a trained clinical research assistant using the REC’s files and electronic archives.

**Results:**

Almost 45% of the reviewed studies (374/822) required clarifications, significant changes to the documentation, or minor changes before final approval.

**Conclusions:**

Preventive measures are needed to reduce the number of requested corrections and thus also the time required for approval, while maintaining review quality. All critical points and proposals presented in this paper require harmonization through updates to European regulations, as regulatory harmonization produces better compliance with rules and reduces the number of changes required before the trials’ final approval. Such updates include the development of standardized formats for informed consent, the verification of any evidence in favor of using off-label treatments over placebo as comparators, using multidisciplinary staff in clinical trials with children and adolescents, improving the legal definition of RECs to assign responsibilities and ensure independence, and providing guidance for RECs to engage clinical research assistants in internal audits.

## Background

A clinical trial (CT) may be initiated if a Research Ethics Committee (REC) and/or competent authority decides that anticipated therapeutic and public health benefits justify the risks and may be continued only if compliance with this requirement is permanently monitored [1]. Institutions, including RECs, are responsible for ensuring compliance with Good Clinical Practice (GCP) [2] and establishing mechanisms to address cases of suspected violations of ethical norms [3].

While many studies have evaluated the structure, process, and outcome of the Institutional Review Board (IRB) review in the United States and have documented inconsistencies and inefficiencies there [4], little research has been conducted on this topic in Italy and other European countries. The aim of this article is to arouse curiosity and further study that compares boards’ activities in Italy, the rest of Europe, and the world. RECs have substantial power and authority over research involving human subjects, and their decisions have substantial implications for those subjects, investigators, and the public at large. However, there is little transparency about RECs processes and decisions [5].

This paper highlights the regulatory issues that Italian RECs might face during the approval phases of CTs. In so doing, it identifies identifying corrective and preventive actions for a more efficient workflow, as well as procedures and structures likely to promote effectiveness and ensuring review quality. The study was conducted in accordance with the ethical principles specified in legally and non-legally binding instruments covering biomedical research related to protocol evaluation during the REC approval phase.

The ethical and scientific standards for conducting biomedical research with humans have been established in international guidelines [2]. Non-legally binding instruments are the Declaration of Helsinki [6], International Ethics Guidelines for Biomedical Research Involving Human Subjects [7], World Health Organization standards, and operational guidance for ethics review of health-related research with human participants, and European Directive 2005/28/EC [8]. These guidelines are compliant with Italian and European rules and help ensure that the dignity, rights, safety, and well-being of participants are protected and that findings are credible.

European regulatory agencies have periodically reviewed these regulations to assess their adequacy and the performance of RECs overseeing CTs. Since RECs play a crucial role in reviewing medical research and protecting human subjects, a quality system is necessary to standardize and continuously improve the flow of activities. The need for regulatory agencies to assess the quality of REC activities led to the measurement of their performance based on the time needed to issue an opinion. Activities related to CTs have also become the subject of detailed legislation in the process of continuous global oversight of medical research [1, 2, 8–10]. In Italy, the competence of a REC impacts the CTs of medicinal products, as well as any other question on the use of medicinal products and medical devices, surgical and clinical procedures, or the study of food products on humans [10].

In Italy, the national Committee for Bioethics (ICB) was established by a decree signed by the President of the Council of Ministers on March 28, 1990, with the task of expressing opinions and the purpose of preparing legislative acts to address the ethical and legal problems that may arise as a result of the progress in scientific research and technological applications on life. In 1992, the ICB emphasized the constitution and legal nature of RECs, their composition, the procedures for appointing the components and any incompatibilities, and the subjects legitimized to establish a REC as unresolved problems [11]. To date, although almost all of these points have been addressed, the legal status of RECs in Italy is not yet expressly regulated. However, according to the interpretation of articles 36–38 and 1228 of the Italian Civil Code, RECs would be similar to an organization, and the responsibility for malicious or negligent facts would be upon those who appointed the Committee and its members [11, 12].

In November 2012, the Italian law n. 189 [10] required all Italian Regions to reorganize their RECs to reduce their excessive number and simplify and rationalize the complicated regulatory framework governing CTs. The highly structured process of coordination and collaboration between sites does not have official guidelines for the standardization of documentation. When it comes to CTs without drugs, harmonizing information is particularly necessary to facilitate the cooperation between the Contract Research Organization (CRO) and RECs. About 90 RECs were operational in Italy at the time of publication [13], which creates significant disparities in the time taken to obtain an opinion on protocols and amendments; moreover, procedures and costs are higher. To better coordinate the studies, a National Coordination Center has been set up to reorganize territorial RECs and reduce their number from 90 to 40 [14].

The present investigation aimed to review protocols that were examined between June 2014 and December 2018 at one of the 90 RECs in Italy to identify corrective and preventive actions that would allow for more efficient review; it further aimed to evaluate the system’s performance to ensure review quality in terms of the subjects’ best interests. Addressing internal discrepancies and identifying areas for improvement is the first important step for IRBs and RECs to follow similar practices.

## Methods

Data were collected from the institutional activity of an Italian REC in Sicily regarding several protocols. For each protocol, the following were collected: trial identification, type of trial, CT phase, evaluation of sample size, use of placebo, number and type of revisions requested by the REC before approval, and time taken for approval. The type of issue was codified to analyze and better identify areas of intervention, according to the following categories: sample size, inclusion criteria, treatment or exam modifications, patient information, legal and administrative modifications, clinical issue, and protocol.

The verification, coding, and validation of data were performed by scientific secretariat staff members who are committed to both preparing the minutes and managing requests to modify the documentation. The number of hours dedicated to each experimental protocol was estimated by dividing the sum of hours committed per year to each working group per the number of evaluations in the same period. The total work was divided into scientific support, administrative support, and reviews conducted by REC members. All data were collected for each experimental protocol by a trained clinical research assistant using the REC’s files and electronic archive. The source data were from the REC’s validated electronic archive, approved meeting minutes, and annual reports on workflow sent to the Regional Bioethics Committee of Sicily.

## Results

The results are summarized as follows. In 53 sessions for 822 opinions on trials, 997 assessments were required with an average of 19 evaluations per Committee session. About 54% (448 of 822) of the protocols were approved with no modifications requested. The percentage of revisions was on average 45.5% (range = 26% to 58% per year; see Table 1). Although only 20 of the 822 protocols were rejected by the REC, 17% of studies required more than one re-evaluation, due to necessary clarifications and significant changes to the documentation, while 34% were approved with minor changes before the final approval.

**Table 1:** Opinions on trials and revision ratio

	2014	2015	2016	2017	2018	Total
Total opinion	61	171	159	224	207	822
Revision	13 of 61 (21.3%)	54 of 171 (31.6%)	74 of 159 (46.5%)	115 of 224 (51.3%)	119 of 207 (57.5%)	374 of 822 (45.5%)
Major revisions	7 of 13 (53.8%)	20 of 54 (37.0%)	59 of 74 (79.7%)	42 of 115 (36.5%)	40 of 119 (33.6%)	168 of 374 (44.9%)
Rejected	0 (0%)	5 of 171 (3%)	10 of 159 (6%)	5 of 224 (2%)	0 (0%)	20 of 822 (2.4%)

Thirty-three protocols required more than two assessments; in seven cases, approval was still conditional on minor modifications, while the remaining 26 received full approval. When revisions were requested, the main reasons were related to legal and administrative modifications, information to the patient and consent modalities, and concerns regarding privacy issues (Table 2). Between May 25 and December 31, 2018, only 6% (4 of 70) needed major revisions regarding personal data processing and the interstate free movement of such data.

**Table 2:** Main reasons for the revisions

Main issues for the revisions	Revised protocols	Ratio (%)
Legal and administrative issues	112	30
Information to the patient and consent	105	28
Privacy	52	14
Sample size	34	9
Protocol	22	6
Treatment or clinical issues	19	5
Inclusion criteria	4	1
Others	26	7
Total	374	100

About 14% of research protocols needed major revisions related to privacy issues (Table 2). After the issuing of European Regulation (EU) 2016/679, only four trials needed revisions related to personal data processing and the free flow of such data.

Drugs were the object of research in 44% of the protocols examined; the remainder concerned clinical trials with medical devices, the use of surgical and clinical procedures, and the study of food products. Of the studies, 60% were international, with a predominance of multicenter studies (80%). Of the randomized controlled trials (RCTs), 77% of the trials that included drugs were for profit and regulatory purposes. In 26% of the protocols with drugs (see Table 3), a placebo was used as a control comparator, and six of these involved children, adolescents, or both. In 41 of 94 placebo-controlled protocol evaluations, documentation integration or clarifications were requested. The same protocol was rejected twice as it involved a placebo arm in a CT related to neurological diseases in pediatric subjects.

**Table 3:** CTs with drugs and comparator placebo usage

Year	CTs with placebo	CTs with drugs	CTs total	% of CTs with placebo (%)	% of CTs with drugs (%)
2014	5	30	61	16.7	49.2
2015	23	78	171	29.5	45.6
2016	17	69	159	24.6	43.4
2017	27	94	224	28.7	42.0
2018	22	88	207	25.0	42.5
Total	94	360	822	26.1	43.8

The REC included 2 scientific staff, 5 administrative staff, and 34 board members. The average workload per protocol was estimated at eight hours in terms of scientific support and assessment of documentation, six hours for administrative support, and seven hours for preliminary evaluation by the referees and evaluation of the opinion on a single protocol. Approximately 160 h per month are needed to perform an improved audit of data retention, dissemination, and publication of results for all approved trials.

## Discussion

This section is divided into the main issues requiring revisions in the reviewed trial. The findings are dealt with separately, and the conclusions are subsequently summarized.

### Legal and administrative issues

These comprise miscellaneous issues that mainly relate to insurance imperfections, which do not raise any ethical concern. The problems encountered concerned either of two matters: the number of experimental centers or insured subjects that did not correspond to those described in the protocol, or the start and end dates of the insurance certificate not being in line with the clinical trial duration.

### Information to the patient and consent

Italian law stipulates that no health treatment can be started or continued unless informed consent of the person concerned is obtained, except in cases expressly indicated [15]. It is important to protect the relationship of care and trust between the patient and doctor based on informed consent, in which the patient’s decision-making autonomy and doctor’s professional competence are preserved [16–18]. The benefit-risk information described in informed consent should reflect the information of the investigator’s brochure and be presented to the patient in concise, simple, objective, and balanced language rather than in a promotional manner [15, 17–19]. The REC requested the integration or modification of informed consent in 105 CTs. Integration was necessary to protect the relationship of care and trust between the patient and doctor based on informed consent, which safeguarded the patient’s decision-making autonomy and the doctor’s professional competence [18–20]. The safeguarding of the rights of individuals who are bound to the patient (i.e., family members and cohabitants) must also be guaranteed [20]. To avoid document integration, adopting an informed consent format including guarantees for the patient and all people involved in the CT could be helpful and avoid any delay in starting the CT, thus reducing expenditures of time and economic resources.

Minors or incapacitated persons have the right to make the most of their understanding and decision-making skills [15, 18, 20]. Participants must receive information on choices concerning their health in a manner appropriate to their ability [21]. Informed consent for the health treatments of a child is given or refused by parents or guardians, considering the child’s will, in relation to his/her age and maturity, having as a goal the protection of mental and physical health by fully respecting his/her dignity. Pediatric CTs are often conducted as multinational trials, and informed consent is part of the documentation submitted for evaluation by the REC. The age of majority is that at which a child acquires full legal capacity, can engage in legal activities, and is liable for any contractual obligations. The majority age is 18 years in all European Union (EU) members except Scotland, where children are considered to have full legal capacity at age 16. In the European Economic Area, 18 years is the legal age of independent consent, with the following exceptions: 14 years in Austria; 15 years in Finland and Denmark; and 16 years in the Netherlands, Ireland, Scotland, and the United Kingdom [19, 22].

In Italy, section four Legislative Decree 211/2003, with regard to the informed consent of children or adolescents in CTs, clarifies that even if a minimum age limit is not set for obtaining informed consent from a minor, he/she should receive information according to his/her capacity of understanding from experienced staff regarding the trial risks and benefits [1]. It is essential to ascertain the explicit wish of a minor capable of expressing an opinion, to refuse participation, or withdraw from a CT at any time [20]. Due to the internationality of many pediatric studies, the REC has decided to suggest the inclusion of a consent form for adolescents older than 12 years if this was not provided. In 17 of the 28 European countries, obtaining consent from school-age children aged 6–8 years is commonly accepted [19, 22]. Problems regarding parents, legal guardians, or children signing the consent form were an issue in 30% of the protocols on children or adolescents. To reduce modifications of protocols, adopting a template for forms allowing the acquisition of consent from school-age children could be helpful.

Even if required by Regulation 536/2014 of the REC, the consent delivered by a minor capable of expressing an opinion should be subsidiary to the informed consent given by the legally designated representative [9]. This seems to be an appropriate integrating point (32) of Regulation 536/2014 to define the age groups for which it is recommended to draw an ad hoc consensus, each of these with age-appropriate language [9, 19–21]. Attached to this Regulation, a template could be produced that researchers could modify as per their trial and submit for review, resulting in reduced approval time and workload for the REC.

### Privacy

The analysis spans a period before and after the European Regulation (EU) 2016/679 issue [23]. Its standard harmonizing effect has been observed in Italy since May 25, 2018, when this legislation was enforced with a drastic reduction in privacy-due revisions’ rate.

The use of personal data is critical to ensure reliability in scientific research. The new Regulation (EU) 2016/679 of April 27, 2016 [23], repealing Directive 95/46/EC, strengthens and harmonizes the rules for protecting individuals’ privacy rights and freedoms. The General Data Protection Regulation (GDPR) is a comprehensive regulation that unifies data protection laws across all EU countries. It defines a set of rights for EU citizens and residents regarding their personal data and enacts strict requirements for companies and organizations on collecting, storing, processing, and managing personal data. Medical trials include the processing of personal health data, genetic data, biometric data, or other kinds of sensitive information, whose use is strictly regulated by the GDPR. Hence, it is necessary to adopt clinical practices compliant with the new EU law. The GDPR introduces new definitions of certain special categories of personal data (such as health, genetic, and biometric data) whose processing is forbidden by principle but permitted for research purposes only in compliance with Articles 9 and 89 of the GDPR. The GDPR is a clear example of how legislation harmonization involves the simplification and reduction of problems related to CT approvals.

### Protocol/treatment or clinical issues

A placebo was used in several protocols involving children and/or adolescents (Table 3) [24]. A CT with pediatric subjects with recurrent forms of multiple sclerosis was submitted twice, and both submissions were rejected for unjustified use of placebo. Reasons for rejection included the increased risk for untreated pediatric subjects in the placebo arm [6, 25, 26]; as for therapeutic indication, in standard pediatric clinical practice, the use of well-established off-label drugs is widespread. The REC’s opinion included the recommendation of a superiority design towards an off-label drug, which was, in that case, standard clinical pediatric practice for the therapeutic indication of the CT.

During the review, many placebo-controlled protocols needed documentation integration or clarifications about placebo use. Ensuring the continuous availability of safe and effective medicinal products authorized for pediatric indications developed in adherence to current regulations is a universal concern [17, 27]. The use of a placebo is typically not ethically controversial when the placebo is compared against an investigational drug in an add-on treatment schedule, or when there is no proven effective treatment for the condition under study [6, 24]. Most medications currently used for the treatment of childhood diseases are either not licensed or prescribed outside the terms of the product license (off-label prescription). Considering children as merely small humans is unacceptable; the major problems related to the formulations and dosage of medicines are often not studied through specific pediatric RCTs [28]. Though many years have passed since the enactment of the European Regulation concerning medicines for pediatric use [29], many pediatric RCTs frequently include a placebo control group to assess the efficacy of new drugs [30]. Many non-patented pediatric drugs, most of which are currently widely used off-label, are under-represented in proposed pediatric RCTs [31, 32]. Guidelines on good pharmacovigilance practices in pediatric populations [18] offer the opportunity to monitor the off-label use of some drugs that in some cases could be considered as consolidated use. During the rejection of protocols that included the use of a placebo for pathologies commonly treated with off-label therapies, the problem was addressed by considering the child’s best interest. The REC argued that well-designed studies, where the efficacy and safety of new drugs are compared with unpatented ones that are currently prescribed off-label would achieve the goal of pediatric regulation in a better and more ethical way than placebo-controlled RCTs. Research on the superiority of new drugs in terms of having better efficacy or safety than the gold standard can avoid the adoption of placebo even if the treatment commonly used was off-label. In this case, there is no need for an arm with placebo, because the study will show if the new drug is better than the treatment considered as the current standard or clinical practice, even if off-label.

Non-inferiority designs require the use of a placebo. When the efficacy of a new drug is proven by comparing it to the gold standard, it must be evident that the lower limit of acceptability does not include the area of activity of the placebo; in other words, even if the new drug is less effective than the reference one, although within accepted limits, it must be superior to the placebo. In a controversial way, the use of a placebo is encouraged by current legislation in the pharmaceutical field; the quality, efficacy, and safety of the new drugs need to be proven but without any need for comparisons with active comparators or any evidence of added value, such as, an increase in efficacy or a decrease in toxicity [33]. The wide off-label use in pediatrics is extensively known, and, generally, scientific literature evidences its efficacy [24–26]. Usually, off-label use is accepted in the patient’s best interest. For example, in Italy, such use is regulated by specific legislation and, in some cases, the National Health System provides the payment for the drug (Law 94/1998 and Law 648/1996). Evidence remains the gold standard to which practitioners should refer for therapeutic decisions for their patients. Given these premises, the integration of European regulations, such as, (EU) N. 536/2014[9] and N. 19 01/2006 [29], is suggested. It is important to promote clear rules concerning the increasingly stringent conditions for which the justified use of placebo should be allowed. RECs should be requested to verify any evidence in favor of off-label treatments to prefer them over placebo as comparators.

### Inclusion criteria/others

Regarding the inclusion criteria and other minor issues, given the limited number of problems and the lack of relevance to the general interest in the questions addressed, they were not considered here for a discussion.

### General comments

- The legal status, composition, function, operations, and regulatory requirements pertaining to RECs are different between EU countries, despite allowing the independent RECs to act in accordance with GCP. A concrete solution would be to indicate, within the European regulations, clinical research assistants (CRAs) as competent figures for the support of the RECs as already envisaged for the CROs [2, 34]. CRAs, in fact, undergo training in risk–benefit evaluation techniques and conduct in-house data safety monitoring to review and assess the risk–benefit ratio and support the REC in this challenging task.
  - A REC can sue/be sued as a third party only if it has a legal personality separate from that of the individual Committee members; however, the legal status of RECs in Italy is not clearly regulated. The Italian decree of July 14, 2009 [35], describes the minimum requirements for insurance policies to protect subjects participating in CTs with drugs. The legal rule and the insurance contract aim to make the experimentation safe, as they allow for the compensation of any damage caused to third parties who voluntarily submit to it. It follows the due protection of people subjected to the experimentation but also the equally dutiful protection of the REC, the sponsor, and the researcher. However, the legislation does not regulate the insurance coverage of members, the Committee, or activities performed in the evaluation or approval or refusal expressed in the performance of their duties. Given the binding nature of the opinions expressed by RECs, to whom would the responsibility be assigned in the event of damage to the patient concerning the decision made?
- To better define the independence of RECs in hierarchical, economical, and functional terms, a better assessment of the legal status of the RECs in Italy and the rest of Europe would be appropriate. This clarification could prevent possible disputes due to the reduced number of RECs compared to the ever-increasing number of health centers that each REC serves. Due to the increasing centralization of experimentation in Europe, it would be appropriate for the regulatory authorities to express their opinion to address this problem. We hope for a regulatory European harmonization to identify the REC as a legal personality and assign responsibilities to it. Such clarifications would be possible through the integration of the regulations issued some time ago and still not fully implemented, (EU) N. 536/2014 [9].
- Although the Italian Legislative Decree of February 8, 2013, Art. 1 paragraph 2 specifies that Italian RECs can also perform advisory functions in ethical issues connected with welfare, to protect and promote a person’s values, this is not in the Europe Regulatory Framework. In the European Regulation, a clearer definition of the field of action for the RECs that is linked to the common clinical practice is necessary. In this context, the RECs could be boards focused on counseling and supporting the exchange of information between medical doctors and patients in typical clinical practices to protect the subjects’ and professionals’ rights. For example, the approval by the REC of informed consent used in standard clinical practice could be made mandatory.
- Even though RECs play a crucial role in reviewing medical research and protecting human subjects, to what degree they fulfill the task they have been assigned is substantially unclear. This results in the call for an evaluation of their activities and, in some places, has led to the establishment of accreditation schemes. At the same time, RECs have become the subject of specific legislation in the process of ongoing global juridification of medical research [9, 10, 36]. Unsurprisingly, there is a tendency to consider the evaluation activity of RECs just as a question of controlling the legal compliance of CTs. However, the ethical considerations led to the genesis of these regulations [37, 38].
- Written policies and procedures will be better assessed in follow-up reviews and monitoring reports of proposed research. This goal can be achieved with the help of CRAs to support RECs in audits on data retention, dissemination, and publication of results to verify compliance with provisions concerning the confidentiality of sensitive data. However, in the European regulations, there is no mention of the need for RECs to acquire CRAs as useful personnel for daily functioning. We suggest that quality support for RECs could be a useful path to attract promoters to clinical centers and, at the same time, emphasize the ethical expertise required by the REC, thus ensuring the patient’s best interest and promoting the ever-needed change in the regulation.

## Conclusions

We wish to indicate that all the critical issues and proposals discussed have, as their common denominator, the need for harmonization through the implementation of the previously mentioned European regulations. As medical standards become more global, there is a need for RECs to follow similar practices.

- The analysis of problems related to informed consent highlights that there are no official guidelines for submissions to RECs, including the necessary documentation and information to be provided. European legislation is needed to harmonize the age groups, for which it is advisable to develop ad hoc informed consent using age-appropriate language. The development of standard formats by the Regulatory Agency could reduce the workflow between the RECs and the CROs that support the promoters of CTs, reducing the time required for final approval.
- We highlighted the problem of CTs with children regarding the unjustified use of placebo and need for scientific evidence for the child’s best interests. By reviewing the study designs/protocols with children to evaluate the efficacy, safety, and off-label use of new drugs, subjects in the control group cannot be treated with a placebo, if the placebo involves suffering, prolongation of illness, or risk or if known effective treatments are available, even if the drug is in consolidated off-label use. Scientific evidence must remain the gold standard; it is important to promote, through the implementation of European regulations, clear rules concerning the increasingly stringent conditions for which the use of a placebo should be allowed. RECs should be requested to verify any evidence in favor of off-label treatments to choose them as comparators over a placebo.
- The involvement of children and adolescents in CTs must always include multidisciplinary staff to support the practitioner in specific aspects of the disease and not consider the pediatric patient as just a tiny human.
- We consider it appropriate to identify the REC as a legal entity to assign it responsibilities and better define its independence in a hierarchical, economical, and functional order. This autonomy would support the possibility of RECs having specialized personnel to better support quality assurance of the services performed. It is important to regulate the insurance coverage of members, committees, and activities performed in the evaluation.
- We ask for support from regulatory agencies in providing guidance so that RECs’ scientific staff can engage CRAs in assisting with audits on data retention and the dissemination and publication of results and to verify compliance with the provisions in force concerning the confidentiality of sensitive data. CRAs could produce in-house safety data to review and assess risk–benefit ratios and support the REC in this challenging task.

As observed in the GDPR case, harmonization produces better compliance with regulations and a reduction in the number of changes necessary for the final approval of the CT by RECs and the time saved, which could emphasize ethical expertise. This need for changes is related to the lack of homogeneity in the regulations in EU countries.

## Data Availability

The source data are in the validated electronic archive of the REC, including approved minutes of the meetings and annual reports on workflow sent to Regional Bioethics Committee of Sicily.

## Abbreviations

CRA: Clinical Research Assistant
CRO: Contract Research Organization
CT: Clinical Trial
DSUR: Development Safety Update Report
EU: European Union
GCP: Good Clinical Practice
GDPR: General Data Protection Regulation
ICB: Italian Committee for Bioethics
IRB: Institutional Review Board
RCT: Randomized Controlled Trial
REC: Research Ethics Committee
