# Correction to: Regulatory, scientific, and ethical issues arising from institutional activity in one of the 90 Italian Research Ethics Committees

**DOI:** 10.1186/s12910-021-00621-7

**Published:** 2021-04-30

**Authors:** G. Benfatto, Regulatory Group, Ethics Committee Catania 1 (Group), F. Drago

**Affiliations:** 1 Clinical Pharmacology and Pharmacovigilance Unit, Regional Pharmacovigilance Center of Catania, G Rodolico-San Marco University Hospital, Catania, Italy; 2 Ethics Committee, Catania 1, G Rodolico-San Marco University Hospital, Via Santa Sofia 78s, 95125 Catania, Italy

## Correction to: BMC Med Ethics (2021) 22:40 10.1186/s12910-021-00605-7

It was highlighted that in the original article [1] the authors of the Regulatory Group1, listed in the Acknowledgements were not tagged in the article XML. The Publisher would like to apologize to the readers and the authors for the inconvenience. The original article has been updated.
